# Microfluidic Paper-Based Blood Plasma Separation Device as a Potential Tool for Timely Detection of Protein Biomarkers

**DOI:** 10.3390/mi13050706

**Published:** 2022-04-29

**Authors:** Francisco Burgos-Flórez, Alexander Rodríguez, Eliana Cervera, Marcio De Ávila, Marco Sanjuán, Pedro J. Villalba

**Affiliations:** 1 Biotechnology Research Group, Universidad del Norte, Barranquilla 081007, Colombia; alexandersanjuan@uninorte.edu.co (A.R.); edcervera@uninorte.edu.co (E.C.); marciod@uninorte.edu.co (M.D.Á.); villalbap@uninorte.edu.co (P.J.V.); 2 Rational Use of Energy and Preservation of the Environment Group (UREMA), Universidad del Norte, Barranquilla 081007, Colombia; msanjuan@uninorte.edu.co; 3 Health and Technological Innovation, Universidad Simón Bolívar, Facultad de Ingenierías, Barranquilla 080002, Colombia

**Keywords:** microfluidics, ELISA, paper, TBI biomarkers, blood plasma separation

## Abstract

A current challenge regarding microfluidic paper-based analytical devices (µPAD) for blood plasma separation (BPS) and electrochemical immunodetection of protein biomarkers is how to achieve a µPAD that yields enough plasma to retain the biomarker for affinity biosensing in a functionalized electrode system. This paper describes the development of a BPS µPAD to detect and quantify the S100B biomarker from peripheral whole blood. The device uses NaCl functionalized VF2 filter paper as a sample collection pad, an MF1 filter paper for plasma retention, and an optimized microfluidic channel geometry. An inverted light microscope, scanning electron microscope (SEM), and image processing software were used for visualizing BPS efficiency. A design of experiments (DOE) assessed the device’s efficacy using an S100B ELISA Kit to measure clinically relevant S100B concentrations in plasma. The BPS device obtained 50 μL of plasma from 300 μL of whole blood after 3.5 min. The statistical correlation of S100B concentrations obtained using plasma from standard centrifugation and the BPS device was 0.98. The BPS device provides a simple manufacturing protocol, short fabrication time, and is capable of S100B detection using ELISA, making one step towards the integration of technologies aimed at low-cost POC testing of clinically relevant biomarkers.

## 1. Introduction

Some pathologies treated in the emergency room or even at the prehospital level are often in need of quick and simple techniques for biomarker detection and quantification so that early decisions that modify medical treatment decisions can be made. In these settings, measurement delays of such biomarkers can lead to a markedly increased risk of fatality or disability. Thus, the development of Point-Of-Care (POC) biosensor systems greatly contributes to timely diagnosis and decision making.

Traumatic Brain Injury (TBI) is one of the pathologies in which this fact is more relevant, because the time window for making timely decisions to establish effective therapies is very short [1]. The treatment of TBI depends on the initial classification of severity, which is classically determined through clinical assessment and head CT, both with reported limitations [2,3,4,5]. In this sense, the advent of the measurement of early biomarkers in blood has been introduced as a more sensitive and cost-effective strategy, among which S100B—a calcium-binding dimeric protein primarily expressed by astrocytes—stands out as a useful predictor of functional outcome in moderate to severe TBI [6].

Previous work from our research group has described the development of electrochemical biosensors for the quantification of S100B in plasma with a benchtop potentiostat [7] and a developed portable potentiostat for POC detection [8]. However, in the clinical context, the measurement of this biomarker demands a technique that is easy to use, readily available, low-cost, and with a fast response time. Unfortunately, the current POC quantification of several biomarkers, including TBI-related biomarkers, requires sample preparation to run assays, which is time-consuming, requires expertise and instrumentation, and represent a high cost [9].

In an idealized POC diagnostic device for TBI-related biomarkers, no sample preparation would be required to run assays [10]. Instead of this, a blood plasma separation (BPS) step is usually needed as pretreatment of the sample for its subsequent use in measuring the analyte of interest, since the blood cellular components (erythrocytes, leukocytes, and platelets) act as a complex dielectric medium that modifies electrochemical interactions during biomarker detection when using electrochemical techniques [11]. Conventional methods such as centrifugation for plasma separation cannot be integrated into a rapid detection platform. Thus, an embedded BPS unit is much more suitable for any kind of POC diagnostics demanding plasma.

Paper-based microfluidic devices (µPADs) have been explored extensively in many applications, one of them being blood plasma separation [12,13,14,15,16,17,18,19,20,21,22,23,24,25]. µPADs offer many advantages over other microfluidic devices made on silicon, glass, or polymer substrates. Among them, they offer high porosity given their cellulose microstructure, which provides high sample volume absorption; are flexible; provide passive fluid transport through capillary action (not requiring external energy sources or instruments); are biocompatible; easily disposable through incineration; and allow cheap, high-throughput fabrication [26,27]. Thus, BPS µPADs provide an ideal platform for designing an inexpensive and efficient POC device and are also ideal for emergencies or remote/developing areas, which are quite common in developing countries and, in general, places with limited resources [25].

Despite having evident attractive characteristics for its use and a considerable number of relevant publications, µPADs have hardly made it into the marketplace [26], as a significant fraction of prototypes remain as a proof-of-concept demonstrations in academic research papers and have yet to prove their usefulness in practical applications. According to Nilghaz et al. [28], the missing keys are the need for complicated user operations, insufficient examination on long-term stability, and reliance on detection equipment unfamiliar for general users.

Several studies have proposed µPADs capable of separating plasma from whole blood samples (Table 1). Plasma separation techniques initially employed anti-A, -B, and -AB antibodies for blood agglutination [14]. Nevertheless, this technique requires expensive and chemically sensitive antibodies, which defeats the purpose of POC. Alternate approaches relying on cell aggregation have been used for plasma separation. Nilghaz et al. [19] developed a paper-based salt functionalized plasma filtration prototype, in which, as blood was added, plasma dissolved the salt and placed the red blood cells (RBCs) in a hypertonic medium, creating an osmotic pressure across the cell membranes, inducing fluid flow towards the outside of the cells and their crenation, giving rise to flat-shaped cells that effectively aggregated, as long as salt concentration was kept below 0.68 M (4% w/v) to not induce hemolysis. Most recent studies have focused on the design and employment of patented separation membranes such as Asymmetric Polysulfone Membrane (APM), Fusion 5, and others to generate lateral and vertical flow filtration [18]. The state-of-the-art also suggests that whole blood volumes should be of at least 100 μL to achieve sufficient plasma yield for further biomarker detection.

Initially, µPADs were typically manufactured by patterning paper with hydrophobic substances or inks, using one of several different printing techniques (such as wax printing and dipping) to define hydrophilic channels and test zones bounded by hydrophobic barriers, commonly made by wax [19,20,22,24,25]. One common limitation to these techniques of patterning paper is that the hydrophobic substances tend to diffuse horizontally in the paper and blur the printed patterns and could account for alterations in fluid flow at the hydrophilic/hydrophobic interfaces, limiting wicking rates during plasma separation [29]. From 2015 onwards, the research has focused on more straightforward techniques by cutting paper into the desired geometry of hydrophilic channels, reducing complexity and cost in device manufacturing.

A current challenge regarding paper-based BPS devices for electrochemical immunodetection of protein biomarkers is how to achieve a µPAD that yields enough plasma to retain the biomarker for further affinity biosensing in a functionalized electrode system. Variability in device manufacturing, assembly, environmental conditions, and sample matrix could significantly impact analyte diffusion from the paper matrix to the electrodes, its binding ability to receptor antibody, and the rate of redox reactions, which could induce higher variance in the measured electrochemical signal. A BPS device that accounts for and diminishes the impact of the former variables would provide more assurance on the accuracy of the BPS tests [10]. In particular, paper filters modified with glass fiber for BPS can sometimes bind analytes on their matrices since hydrophobic interactions can occur between proteins and the highly hydrophilic glass fibers. One way to avoid this has been to coat paper-based BPS devices with polymers that make their matrix more hydrophilic, such as Polyvinyl alcohol (PVA), which can provide highly hydrophilic matrices that do not interact with most analytes [18].

Considering the above, BPS devices aimed at POC should use simple manufacturing techniques such as paper cutting, overlapping, and coupling for shaping hydrophilic channels, forgoing wax printing techniques. Similarly, BPS should be performed by integrating various separation techniques such as aggregation and membrane lateral–vertical filtration, which can provide a synergetic effect on the separation efficiency, and the use of design of experiments (DOE) could optimize the device materials and geometry to allow adequate plasma volumes with low separation times for subsequent biomarker detection, robust enough for its employment in clinical settings.

To our knowledge, no studies regarding paper-based BPS devices for the pretreatment of peripheral blood samples to detect TBI-related biomarkers have been published. Therefore, this work describes the development of a passive, microfluidic paper-based BPS device with the potential to integrate to a POC system for detection of a protein biomarker in peripheral blood samples, such as the S100B protein. The device design is based on the salt functionalization technique [19], with microfluidic channels to allow capillary force to move the whole blood sample while retaining cellular content [20]. Final device assembly uses simple paper cutting and assembly techniques for reducing manufacturing variability and complexity [17,18]. The developed BPS µPAD is made up of a NaCl-modified VF2 bound-glass fiber filter paper used as a sample collection pad and an MF1 filter paper for plasma retention, which together can yield 50 μL of plasma after 3.5 min from 300 μL of whole blood added on the VF2 sample collection pad. An inverted light microscope, a scanning electron microscope (SEM), and an image processing software were used for assessing plasma separation efficiency. Plasma flow over time was modeled by measuring separated plasma volume in the MF1 pad, and a DOE assessed the device’s efficacy using an S100B ELISA Kit to measure S100B concentration in the separated plasma absorbed by the MF1 pad.

The proposed device provides a simpler manufacturing protocol than previous BPS devices found on the literature, with reduced fabrication times and the ability to separate plasma for the detection of the S100B biomarker using ELISA, making one step towards the integration of technologies aimed at low-cost POC testing of clinically relevant biomarkers.

## 2. Materials and Methods

### 2.1. Reagents and Manufacturing Materials

An S100B SimpleStep ELISA^®^ Kit (ab203359) was purchased from Abcam, UK. Deionized water (MiliQ^®^) was employed to prepare all solutions. NaCl solutions of 0.68 M were prepared using analytical grade NaCl (Sigma-Aldrich). MF1 and VF2 glass fiber filter papers were purchased from Sanitas Lab technology.

### 2.2. Blood Sample Preparation

Human whole blood samples with 45% Hematocrit content were obtained from a venipuncture of a healthy donor, collected in Gel & EDTA K2 tubes (Improvacuter^®^ 722350202), and used within two hours for the experimental runs (Ethics Committee, act 167, Universidad del Norte, 25 January 2018). All experiments were performed with the same donor blood to maintain unified experimental conditions. Low retention tubes were employed to make 500-μL aliquots, which were then spiked with corresponding amounts of the S100B protein up to the desired concentrations between 316, 562, and 1000 pg/mL, and vortex mixed for one minute. Plasma was extracted either by centrifugation of aliquots for 30 min at 10,000× *g* followed by pipetting and aliquoting in low retention tubes, or by using the BPS device.

### 2.3. Device Design and Fabrication

**Figure 1:**
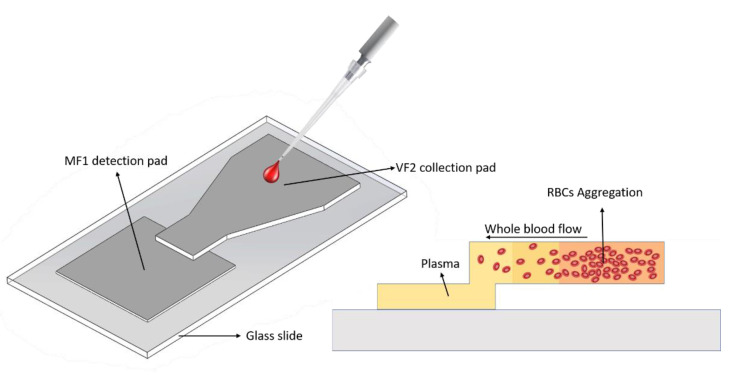
Schematic representation of the paper-based BPS device. The whole blood sample is add-ed to the VF2 collection pad, which performs vertical and lateral plasma separation. The addition of NaCl to the VF2 pad induces RBCs aggregation and higher plasma wicking as cells crenate and become stuck in the VF2 matrix due to hypertonic conditions.

The fabrication of the proposed BPS device was aimed to be as simple as possible to allow users fast optimization of the design for their purposes and needs (see Figure S1). The device comprised two zones: a whole blood collection pad in charge of plasma separation, and a plasma detection pad where the separated plasma was accumulated and later used for S100B biomarker detection (Figure 1). Initially, a cardboard mold was fabricated with the geometry dimensions of the proposed BPS device. A piece of VF2 polyvinyl alcohol-bound glass fiber filter paper was cut into a rectangle of dimensions 1.7 cm × 2.7 cm. Using a craft cutter, the VF2 rectangle was shaped to the geometry of the cardboard mold. Next, a 1.7 cm × 1.7 cm square piece of MF1 polyvinyl alcohol-bound glass fiber filter paper was cut for the detection pad. MF1 paper was placed on a glass slide, and the VF2 paper was horizontally aligned and placed on top of it with approximately 3 mm of overlap. A conventional 2 mm-slice of hydrophobic paper tape was attached to the narrow VF2 channel right before VF2-MF1 surface overlap and to the glass slide for fixing the BPS device. A total 150 μL of 0.68-M NaCl solution was slowly dropped on the VF2 collection pad, which was then dried for 15 min at 37 °C in an oven. The BPS device dimensions were optimized to allow 300 μL of sample addition to the VF2 collection pad and 50 μL of separated plasma in the MF1 detection pad. Finally, the triangular narrowing geometry of the VF2 microfluidic channels was defined to provide a uniform flow towards the detection pad.

Plasma separation on the proposed device is achieved by means of three mechanisms: Osmotic pressure, microfluidic channel size reduction, and cell trapping by the filter membranes. The functionalization of the VF2 pad with the NaCl solution generates a hypertonic medium that induces an osmotic pressure on RBCs, which lose water and shrink. In these conditions, RBCs aggregate by forming closely packed deflated disks that separate from the plasma, which continues to wick along the filter paper membranes. The reduction in channel size in the VF2 pad before the VF2–MF1 overlapping interface induces higher fluid flow, while its pore size allows removal of particles greater than 2–3 mm including RBCs and platelets.

### 2.4. Separation Efficiency Analysis

Five μL of whole blood or separated plasma were added between two 0.5 mm-thick microscope slides and observed in a Zeiss Axio Observer z1 inverted microscope (Zeiss, Oberkochen, Germany). The separation efficiency of the BPS device was initially qualitatively evaluated by comparing images at 40× of the whole blood samples and the plasma samples obtained after separation with the developed device. Similar to [18], the quantitative separation efficiency of the BPS device was defined as the difference between the number of RBCs in whole blood vs. the number of RBCs in the separated plasma, defined as

(1) $$Se=1-\frac{N_{p}}{N_{w}}$$

where $N_{p}$ corresponds to the number of cells counted in plasma and $N_{w}$ is the number of cells counted in whole blood. RBC count was performed by taking pictures of the 40× amplified images and using the Java image processing software ImageJ, which allowed the segmentation of circular figures corresponding to RBCs in either plasma or whole blood. This process was performed on five whole blood samples and five plasma samples obtained with the BPS device to capture the mean and standard deviation of the BPS device separation efficiency. The separation efficiency of the BPS device was also qualitatively assessed using a JEOL JSM-5600 SEM with 5 KV of accelerating voltage. The observations of the formed elements of blood on the surface of the VF2 collection pad and the MF1 detection pad were made after whole blood samples were filtered with adding and drying of the 0.68 M NaCl solution on the VF2 surface as previously described.

### 2.5. Plasma Flow over Time

Paper-based capillary-driven flow along the paper interfiber passages is usually not constant, as it depends on the capillary inhibition caused by the randomly distributed cellulose fibers and other components forming the paper matrix [15]. Hence, to model separated plasma volume over time, BPS device collection pads were weighted every 20 s to measure plasma content after initial sample addition. Three BPS devices were used for each data point to provide enough statistical power to the assay (see Table S1). Plasma volume was calculated using a reference density of 1.025 g/mL for blood plasma.

### 2.6. Blood Sample Volume Optimization

To optimize sample volume for extracting 50 μL of plasma, whole blood samples at 225, 250, 275, 300, 325, and 350 μL were applied onto VF2 collections pads. The time until plasma filled the detection zone was recorded as well as the presence of blood saturation and overflow in the collection pad and whole blood passing to the MF1 detection pad.

### 2.7. S100B Detection Mechanism

An S100B SimpleStep ELISA^®^ Kit (ab203359) was used to quantify S100B in human plasma samples. All operations were strictly performed according to the manufacturer’s instructions (sensitivity of 139 pg/mL, range of detection between 0.31 ng/mL and 20 ng/mL). A total 200 μL of whole blood samples containing S100B at 0, 316, 562, and 1000 pg/mL were centrifuged at 4 °C at 3000 rpm for 30 min. Fifty μL of the supernatant were collected from each centrifuged sample and added to the defined ELISA microplate wells. A total 300 μL of whole blood samples containing the same S100B concentrations were applied to the BPS device collection pads. After 3.5 min, BPS device detection pads were gently squeezed to obtain 50 μL of plasma, which were then added to the appropriate wells of the ELISA microplate. A Synergy™ 2 Multi-Detection Microplate Reader was used to measure absorbance at 450 nm.

### 2.8. Design of Experiments

A DOE was performed to assess the detection of S100B by using the BPS device for plasma obtention compared to plasma obtained by centrifugation. Considering the ELISA test sensitivity, the biomarker S100B was detected in whole blood concentrations in three levels set in a logarithmic scale, using concentrations with clinical utility: 316 pg/mL (log₁₀ = 2.5), 562 pg/mL (log₁₀ = 2.75), and 1000 pg/mL (log₁₀ = 3). The optical density (OD) minus signal background was selected as the response variable. Eight experimental runs were used for obtaining the calibration curve of the ELISA assay. In addition, three randomized experimental runs (*n* = 3) were made for each separation method at each S100B concentration, and three additional runs were performed with 0 pg/mL S100B whole blood samples for the BPS device, giving a total of 29 experimental runs.

### 2.9. Robustness Evaluation against Disturbance Factors

An HTC-1 LCD Digital Thermometer Hygrometer (HTC Instruments, Mumbai, India) was employed to measure temperature and humidity on each experimental run and test whether there was a significant linear correlation between these variables and the OD obtained with the ELISA kit at each biomarker concentration.

### 2.10. Statistical Analysis

Statistical analysis was performed using RStudio and Statgraphics Centurion 18. Statgraphics was employed for finding a regression model together with a lack of fit test from the plasma flow over time, and the ELISA standardization runs to validate the tests. Model suitability was also established considering global model significance, coefficient significance, and analysis of residuals structure. A statistical T-test was performed between the OD values from the ELISA test of plasma samples obtained from the developed plasma separator and those obtained by centrifugation for each defined S100B concentration. The assumptions of normality, homoscedasticity, and independence of residuals were assessed to establish the statistical validity of the T-tests. Finally, Pearson’s correlation coefficient was calculated in RStudio to check for linear correlation between OD values, and temperature and humidity. All statistical tests were deemed significant with a *p*-value lower than 0.05.

## 3. Results and Discussion

This paper describes the development of a paper-based BPS device for S100B detection using an ELISA kit. The manufacturing methods and surface modifications have been designed for easy and highly repeatable construction, taking into consideration previous works reported in the literature [17,19,20].

### 3.1. Device Fabrication

**Figure 2:**
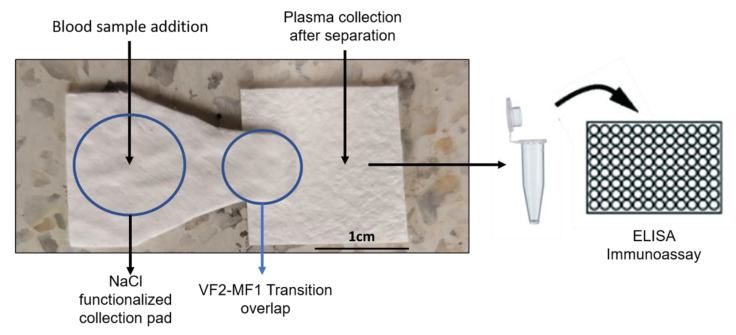
Fabricated BPS device. The sample is added to the center of the NaCl-functionalized VF2 collection pad and wicks towards the MF1 plasma reservoir for S100B detection on the ELISA immunoassay.

The fabricated paper-based BPS device with 595 mm^2^ of total separation area is shown in Figure 2. A two-part BPS was obtained by combining a NaCl-functionalized VF2 collection pad with an MF1 detection pad. In future works, geometry scaling can be made if lower amounts of plasma are needed and, therefore, less sample volume will be required for effective separation.

### 3.2. Separation Efficiency Analysis

**Figure 3:**
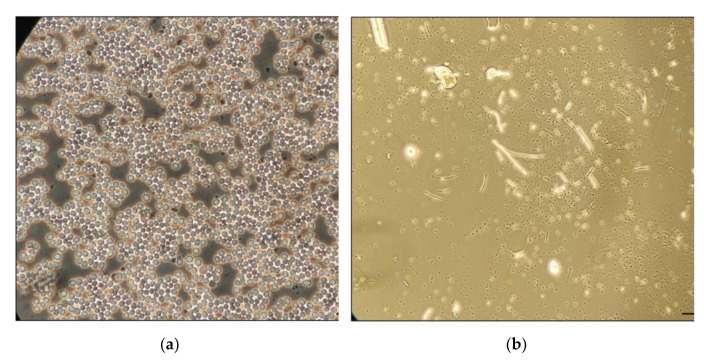
Visual comparison of RBCs content in whole blood versus plasma separated with the paper-based BPS device. (**a**) Whole blood sample. (**b**) Plasma sample from BPS device.

The qualitative evaluation of separation efficiency showed a lower RBC count in the filtrate than in whole blood (Figure 3), as few cells could be seen on the separated plasma. The quantitative separation efficiency using cell segmentation and counting by image processing techniques showed an average efficiency higher than 95% with a standard deviation below 4% (Table 2), demonstrating that the BPS device could effectively perform RBC capture along the VF2 collection pad volume for yielding separated plasma in the MF1 detection pad.

**Figure 4:**
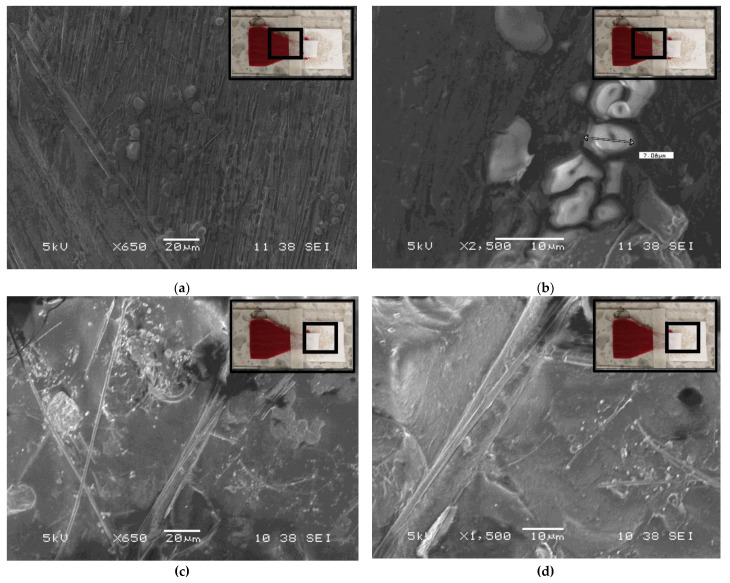
Visual comparison of SEM images of the remaining formed elements of a whole blood sample on the surface of the VF2 collection pad at (**a**) 650× and (**b**) 2500×, and on the MF1 detection pad at (**c**) 650× and (**d**) 1500×. RBCs bound to the VF2 paper fibers are shown in (**b**), and the diameter of one RBC is displayed.

The SEM observations exhibited a lower number of blood cells inside the fibers of the MF1 detection pad compared with the number observed on the VF2 collection pad (Figure 4). Blood cells become trapped on the surface of the VF2 collection pad at 650× (Figure 4a) and 2500× (Figure 4b), while smaller particles become trapped within the fibers of the MF1 detection pad at 650× (Figure 4c) and 1500× (Figure 4d). These findings indicate that the BPS device operates as a gradual filter by trapping blood cells on the surface of the first pad (VF2) and the smaller formed elements of blood into the micrometric spaces between the fibers of the second pad (MF1). This property represents an efficient mechanism to reduce the clogging of the BPS device, facilitating rapid filtration.

Since plasma flow along the BPS device is driven by capillary forces, the incorporation of the MF1 detection pad, which could hold up to 60 μL before saturation, is a way to control sample wicking and plasma volume as it inhibits diffusion by reducing capillary pressure once it becomes saturated. The VF2 paper provided quick plasma flow and high RBC aggregation along its length and near the sample deposition site while maintaining low RBC hemolysis and overflow to the MF1 detection pad. Even though the separation efficiency of the developed BPS device was lower than the 99% efficiency obtained by Lu et al. [18], it was still sufficient for the purposes of this work, given its low cost, easy fabrication, and high plasma yield.

### 3.3. Plasma Flow over Time

**Figure 5:**
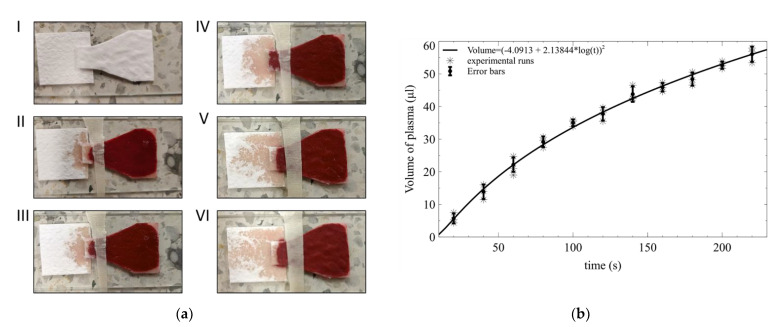
Blood plasma separation using the developed BPS device. (**a**) A total 300 μL was applied to the collection pad. Separation at (I) t = 0 s; (II) t = 40 s; (III) t = 85 s; (IV) t = 125 s; (V) t = 170 s; (VI) t = 210 s. (**b**) Volume of separated plasma as a function of time.

The S100B detection ELISA test requires consistent sample volumes for achieving precise and accurate measurements of the biomarker. Hence, adequate control of separation yield is a requisite for achieving a successful test. The MF1 detection pad of the BPS device could separate more than 50 μL after 220 s of adding the whole blood samples to the VF2 collection pad. Figure 5a shows the process of BPS at different times after the 300 μL of whole blood samples are placed on the VF2 collection pad of the BPS device. Separation is achieved with minimal RBC breakthrough to the MF2 detection pad.

A regression model was found for the volume of separated plasma as a function of elapsed time (Figure 5b). Three experimental runs for each time point were made in a randomized order. Each point on the regression model represents each independent measurement, and the error bar represents the standard error of the mean. The model was found suitable for the experimental results since its coefficients were significant, no structure was found on its residuals, and the lack of fit test was not significant (Figure S2 and Table S2). Overall, the BPS device can yield 78% after 2 min and over 94% after 3.3 min.

### 3.4. Blood Sample Volume Optimization

**Figure 6:**
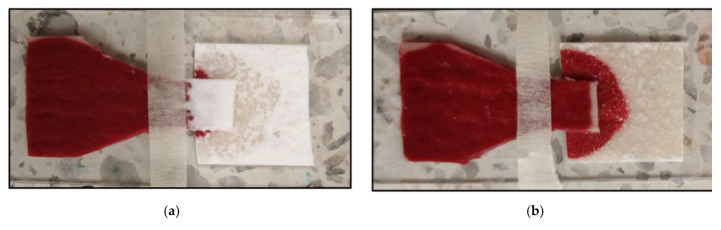
The behavior of BPS device with whole blood volumes below and above 300 μL: (**a**) 275 μL of whole blood; (**b**) 325 μL of whole blood.

Several experimental runs were made to assess the BPS device limits in applied blood sample volume to achieve effective plasma separation without RBC overflow. Using volumes equal to or lower than 275 μL did not yield high enough quantities of plasma for ELISA S100B detection (Figure 6a). The addition of volumes of whole blood higher than 325 μL induces RBC overflow towards the MF1 paper (Figure 6b), impeding plasma recovery for further S100B detection in the ELISA wells. Therefore, all further experimental runs for S100B detection were made with 300 μL of applied whole blood samples in the VF2 collection pads of the BPS device.

### 3.5. S100B Detection

**Figure 7:**
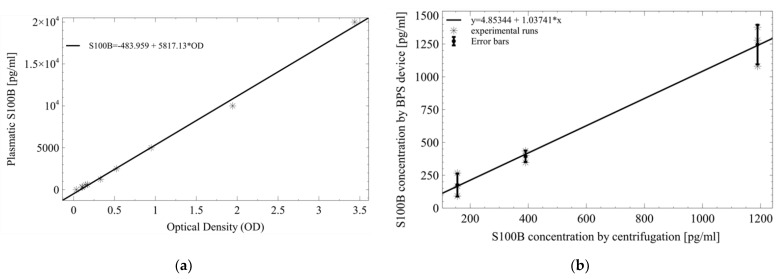
Regression models for the ELISA test. (**a**) Calibration curve for standard ELISA measurements. (**b**) Correlation model for S100B detection values obtained using centrifugation and the BPS device.

S100B concentration was detected by measuring the OD of each experimental run according to the ELISA well plate design (Table S3). A calibration curve for the standardization of the ELISA test is shown in Figure 7a. The model was suitable for the experimental results since its coefficients were significant, and no structure was found on its residuals (Figure S3 and Table S4). The correlation between S100B concentrations using plasma obtained from standard centrifugation and the BPS device was equal to 0.98031 (Figure 7b), suggesting that the BPS device could be used as an alternative for plasma separation in the conditions defined for this work. Environmental factors (temperature and humidity) were also measured during plasma separation by both methods. No significant correlations (Table S5) were found between OD and temperature (*p*-value 0.88, Correlation coefficient 0.048) and OD and humidity (*p*-value 0.80, Correlation coefficient −0.078) from separated plasma using the BPS device for the given experimental conditions. Considering that temperature and humidity variations could induce sample evaporation and viscosity changes during BPS, their effect on the assay could be significant and detrimental to the device performance in environmental conditions different from the ones used in this work.

Statistical T-tests were carried out between the S100B concentration results obtained with conventionally separated plasma and plasma coming from the BPS device at each defined S100B concentration (Table 3, Table 4 and Table 5). The standardized skewness for each T-test was within the (−2,2) range; so, samples could be considered to come from normal distributions. No significant differences were found in the sample variances using F tests, and no structure was found in the residual plots (Figure S4). Considering that the T-test *p*-values were higher than 0.05, the null hypothesis could not be rejected. Thus, there were no statistically significant differences between the means of the two samples at each evaluated S100B concentration. These results suggest that the BPS device could be used as an alternative to centrifugation in the experimental conditions defined in this work.

POC testing devices using whole blood often demand low volume samples to permit fast collection with fingersticks. Albeit the BPS design was optimized for separating up to 50 μL of plasma, geometric adaptations could be easily achieved to yield lower amounts of separated plasma from lower whole blood sample volumes (lower than 200 μL) for POC detection of biomarkers by electrochemical techniques that employ microfluidic detection wells with less than 20 μL of volume. Still, the volume of 300 μL was found to be optimal for BPS, as it allowed MF1 plasma filling with minimum RBC leakage and provided consistent plasma volumes for further S100B detection through ELISA.

Plasma yield of the proposed device was close to the maximum value in the range reported by [13,14,16,18], as other studies did not report results about this metric. The overall separation time of 220 s stayed near the minimum value of the range reported by previous studies in Table 1, while the efficiency of 95% was near the maximum reported value. Even though the device performance was not significantly higher on any given metric, its overall performance was higher than previous works.

To our knowledge, no studies have been reported about µPADs for BPS in a detection scheme of TBI-related protein biomarkers. Thus, the most important contribution of this work is its validation as a sample pretreatment step in the detection of S100B in peripheral blood. Hence, the proposed BPS device improves the previously reported technologies by optimizing their overall capabilities in an easily manufactured device that could be integrated in future low-cost POC biomarker detection systems.

### 3.6. Limitations

Various limitations should be discussed for the developed BPS device if optimization of the technology for commercial applications is desired. Initially, the BPS devices developed in this work were tested with whole blood samples from only one donor and spiked with clinically relevant S100B concentrations. The effect of hematocrit percentage and the use of a statistically sufficient number of diverse TBI patient’s whole blood samples containing a wide range of S100B concentrations should be the next step for assessing the developed BPS device. Similarly, identifying the effect of nuisance factors, such as temperature and humidity, in BPS and subsequent S100B measurements on the developed prototype must be established in real settings beyond the laboratory. In addition, the effect of the disruption of the BBB and other tissue alterations related to TBI and other conditions should be assessed as it could change blood content and affect the BPS and the subsequent measurement of the biomarker. Sample evaporation should also be considered and mitigated by encapsulating the BPS device in future prototypes. The demonstration of successful S100B detection in the proposed conditions using ELISA or alternate protein detection and quantification techniques could be a step towards maturing the technology for its use in POC Testing (POCT), where conventional plasma obtention through centrifugation cannot be performed. Hence, further studies using plasma samples from multiple individuals presenting variable degrees of TBI and hematocrits to evaluate and optimize the BPS device performance should be performed. More detailed studies are currently underway to test the BPS device with the above considerations.

## 4. Conclusions

The guidelines for effective POCT devices provided by the World Health Organization (WHO) are summarized under the "ASSURED" acronym, which stands for Affordable, sensitive, specific, user-friendly, rapid and robust, equipment-free, and delivered (accessible to end-users) [30]. This work has advanced the state-of-the-art of POC testing of TBI protein biomarkers such as S100B by developing a microfluidic paper-based BPS device that allows fast, affordable, equipment-free plasma obtention from whole blood samples to detect S100B using an ELISA kit, forgoing the need for expensive plasma separation processes using conventional centrifugation. The integration of paper cutting techniques, geometrical optimization of microfluidic channels, and NaCl functionalization in a simple device allowed the manufacturing of a simple BPS device capable of fast BPS (less than 4 **min** after whole blood was added to the sample collection pad), providing a user-friendly platform that could be optimized to achieve a wide range of plasma volumes aligned to the biosensing platform requirements and user demands for other applications beyond the scope of S100B POC detection for TBI diagnosis support and treatment.

## Figures and Tables

**Table 1:** Passive paper-based BPS prototypes found in the literature.

Reference	Capillary Action	Fabrication	Paper Types	Plasma Yield (%)	Separation Time (s)	Hematocrits in the Sample (%)	Blood Volume (μL)
Songjaroen (2012) [22]	Vertical and lateral flow by membrane separation	Wax dipping	MF1 and LF1 and Whatman No. 1	N.R.	100–200	24–55	8–20
Vella (2012) [24]	Vertical flow by membrane separation	Wax printing	VF1, VF2, MF1, Fusion 5, GX, GR, GF, and Whatman No. 1	N.R.	300–600	N.R.	10–20
Yang (2012) [25]	Vertical and lateral flow by agglutination with anti-A, -B, and -AB antibodies	Wax printing and antibody drop-casting	Whatman No. 1	N.R.	<300	N.R.	7
Noiphung (2013) [20]	Vertical and lateral flow by membrane separation	Wax dipping	VF1, VF2, and Whatman No. 1	N.R.	170–270	24–60	200
Gong (2013) [13]	Vertical and lateral flow by membrane separation	Laser micromachining of PMMA	Polysulfone asymmetric membrane GR VIVID	6–35	300	N.R.	800–1000
Kim (2013) [16]	Vertical and lateral flow by membrane separation	N.R.	NOVIPLEX separation membrane	12–30	180	20–71	25
Nilghaz (2015) [19]	Lateral flow controlled by red blood cells aggregation	Wax patterning	NaCl functionalized Whatman No.4	N.R.	180	35–55	3–10
Kar (2015) [15]	Lateral flow controlled by H channel and changes in blood rheological properties	Inkjet printing followed by Origami protocol	Whatman No. 4 and PBS solution	N.R.	200	37	50
G. Li (2015) [17]	Vertical and lateral flow by membrane separation	Paper cutting	Polysulfone asymmetric membrane GR VIVID, Fusion 5 y 10G surfactant treated nitrocellulose membrane	N.R.	180	40	15–40
Robinson (2016) [21]	Vertical and lateral flow by membrane separation	Paper cutting	Polysulfone asymmetric membrane GR VIVID, glass fiber membrane filter, and nitrocellulose membrane	N.R.	360	N.R.	40
Tiwari (2017) [23]	Vertical and lateral flow by functionalized membrane separation	Paper cutting	GF/C glass fiber paper disks, Whatman No.1, No.2, No.5, and Zinc nanorods	N.R.	600	N.R.	3
Bhamla (2017) [12]	Paper-based centrifuge	Paper cutting	Paper wood, Nylon threads	N.R.	90	N.R.	20
Lu (2018) [18]	Vertical and lateral flow by membrane separation	Papercutting bound with adhesive tape	Polysulfone asymmetric membrane GR VIVID, FR-1 filter pad, Fusion 5	20–30	600	30–60	50–70
Guo (2020) [14]	Vertical and lateral flow by agglutination with anti-A, -B, and -AB antibodies	Paper cutting	N.R. Synthetic paper	11	316	45	90
This work	Vertical and lateral flow by membrane separation and RBC agglutination	Papercutting bound with adhesive tape	MF1, NaCl functionalized VF2	30	220	45	300

N.R: Not reported.

**Table 2:** The separation efficiency of RBCs using the BPS device.

Experimental Run	Number of RBCs in Whole Blood	Number of RBCs in Separated Plasma	Separation Efficiency
1	1208	99	0.92
2	983	35	0.96
3	1147	78	0.93
4	947	24	0.97
5	1369	18	0.98
**Mean**	1130.8	50.8	0.95
**Standard deviation**	172.2	35.73	0.03

**Table 3:** Statistical comparison of conventional and paper-based blood plasma separation for measuring S100B at 316 pg/mL using ELISA.

	S100B Concentration Using ELISA	
Experimental Run	Centrifugation	BPS Device
1	138.47	167.56
2	126.84	266.45
3	202.46	91.93
**Mean**	155.92	175.31
**Standard deviation**	40.71	87.51
**Skewness**	1.11	0.27
	**Test**	***p*-Value**
**F-test** $H_{0}:\;\sigma_{1}=\sigma_{2},\;\alpha =0.05$	4.61	0.35
**T-test** $H_{0}:\;\mu_{1}=\mu_{2},\;\alpha =0.05$	0.34	0.74

**Table 4:** Statistical comparison of conventional and paper-based blood plasma separation for measuring S100B at 562 pg/mL using ELISA.

	S100B Concentration Using ELISA	
Experimental Run	Centrifugation	BPS Device
1	371.15	400.24
2	417.69	435.14
3	382.79	347.89
**Mean**	390.54	394.42
**Standard deviation**	24.21	43.91
**Skewness**	0.91	−0.41
	**Test**	***p*-Value**
**F-test** $H_{0}:\;\sigma_{1}=\sigma_{2},\;\alpha =0.05$	0.30	0.46
**T-test** $H_{0}:\;\mu_{1}=\mu_{2},\;\alpha =0.05$	−0.13	0.89

**Table 5:** Statistical comparison of conventional and paper-based blood plasma separation for measuring S100B at 1000 pg/mL using ELISA.

	S100B Concentration Using ELISA	
Experimental Run	Centrifugation	BPS Device
1	1237.91	1377.52
2	1191.37	1080.85
3	1139.02	1278.63
**Mean**	1189.43	1245.67
**Standard deviation**	49.47	151.05
**Skewness**	−0.12	−0.66
	**Test**	***p*** **-Value**
**F-test** $H_{0}:\;\sigma_{1}=\sigma_{2},\;\alpha =0.05$	0.10	0.19
**T-test** $H_{0}:\;\mu_{1}=\mu_{2},\;\alpha =0.05$	−0.61	0.57

## Supplementary Materials

The following supporting information can be downloaded at https://www.mdpi.com/article/10.3390/mi13050706/s1, Figure S1: CAD geometry designs of BPS device papers; Table S1: Experimental runs for plasma volume over time; Figure S2: Analysis of the structure of the residuals for the regression model of separated plasma volume over time; Table S2: Suitability analysis and Lack-of-fit test of plasma volume over time regression model; Table S3: S100B concentration, Optical density, temperature, and humidity values for each experimental run of the ELISA; Figure S3: Analysis of the structure of the residuals for the standard S100B detection model using ELISA; Table S4: Suitability analysis of the ELISA S100B detection standard model; Table S5: Pearson’s correlation between OD and temperature and humidity; Figure S4: Analysis of the structure of the residuals for the t-test of conventional and paper-based BPS runs for measuring S100B using ELISA.
