# Supplementary material for: Microfluidic Paper-Based Blood Plasma Separation Device as a Potential Tool for Timely Detection of Protein Biomarkers
Source: Micromachines (Basel). 2022 Apr 29;13(5):706. doi: 10.3390/mi13050706 (PMC9142996; doi:10.3390/mi13050706)

S1. Electronic supplementary information

Figure S1. CAD geometry designs of BPS device papers. A) VF2 collection pad. B) MF1 detection pad. Dimensions are in millimeters.

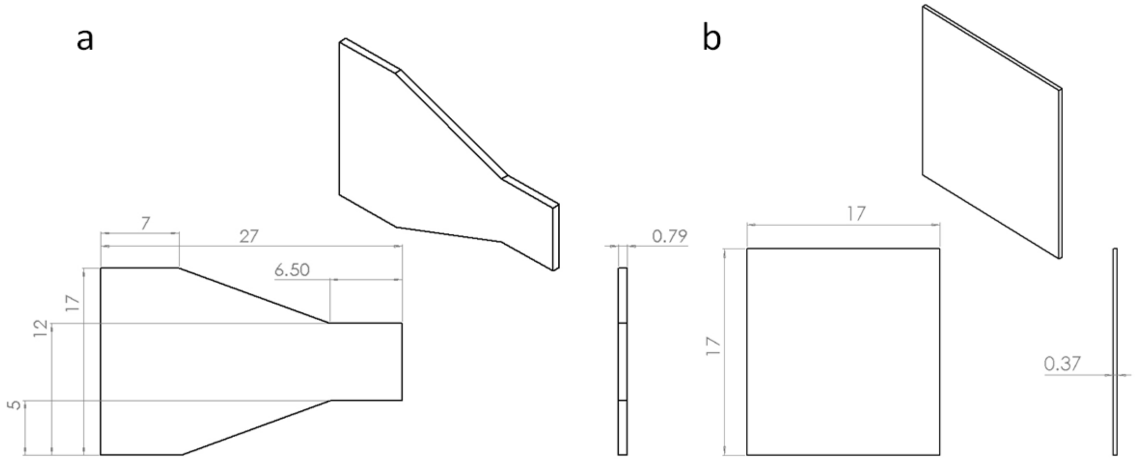

Table S1. Experimental runs for plasma volume over time.

time	volume (μl)
120	38.14
60	21.26
160	47.31
180	48.19
40	11.60
180	50.53
160	44.68
180	46.53
80	30.63
220	56.78
200	52.58
100	35.80
60	24.68
80	29.56
200	51.60

80	27.41
220	53.46
100	35.41
120	35.51
120	39.60
20	5.36
220	57.95
40	13.95
140	42.53
60	20.58
100	33.85
140	46.53
200	53.85
20	4.39
40	16.09
20	7.41
160	45.75
140	42.34

Figure S2. Analysis of the structure of the residuals for the regression model of separated plasma volume over time.

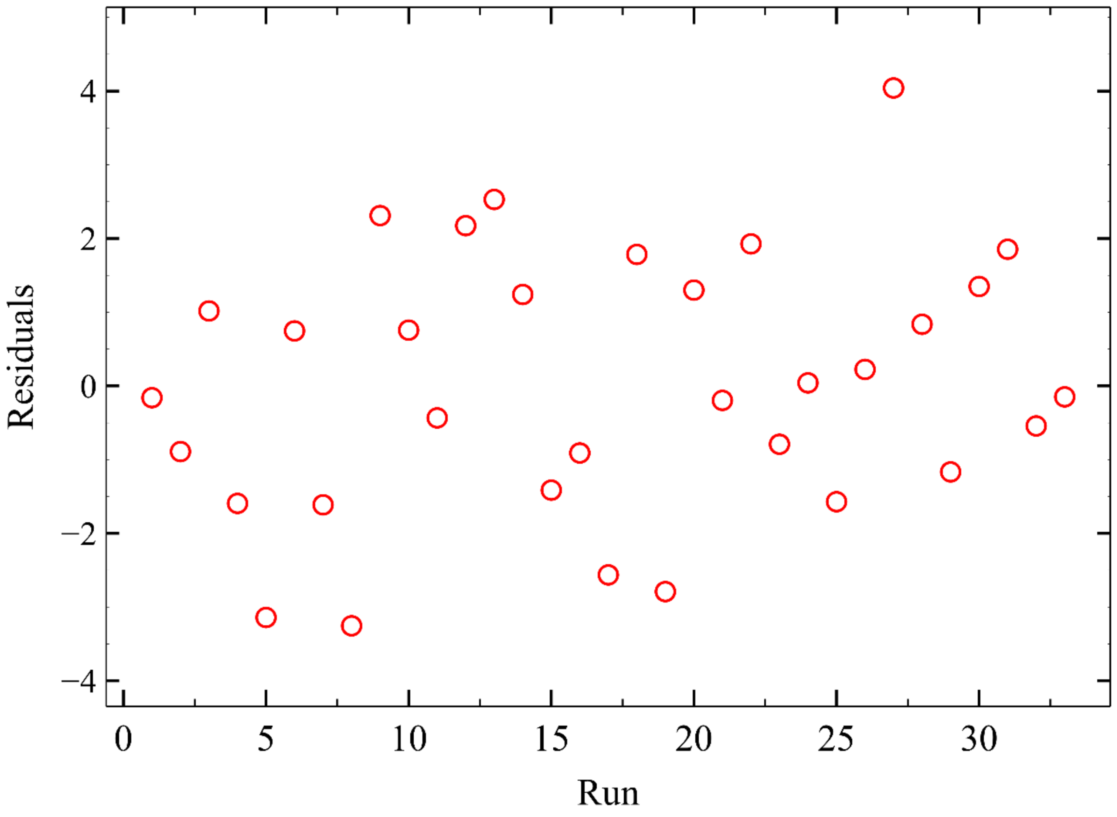

Table S2. Suitability analysis and Lack-of-fit test of Plasma volume over time regression model.

Coefficients

Parameter	Least Squares Estimate	Standard Error	T Statistic	P-Value
Intercept	-4.09127	0.21332	-19.1872	0.0000
Slope	2.14627	0.04593	46.7197	0.0000

Analysis of Variance

Source	Sum of Squares	Df	Mean Square	F-Ratio	P-Value
Model	76.7240	1	76.7240	2182.73	0.0000
Residual	1.08966	31	0.0351505		
Total (Corr.)	77.8137	32			

Correlation Coefficient = 0.992974

R-squared = 98.5996 percent

R-squared (adjusted for d.f.) = 98.5545 percent

Standard Error of Est. = 0.187485

Mean absolute error = 0.14312

Durbin-Watson statistic = 1003617 (P=0.5508)

Lag 1 residual autocorrelation = -0.0181408

Analysis of Variance with Lack-of-Fit

Source	Sum of Squares	Df	Mean Square	F-Ratio	P-Value
Model	76.7240	1	76.7240	2182.73	0.0000
Residual	1.08966	31	0.0351505		
Lack-of-Fit	0.194738	9	0.0216375	0.53	0.8357
Pure Error	0.894927	22	0.0406785		
Total (Corr.)	77.8137	32			

Table S3. S100B concentration, Optical density, temperature, and humidity values for each experimental run of the ELISA.

Separation method	S100B (pg/ml)	Temperature (°C)	Humidity (%)	Optical Density	S100B
Centrifugation	0	24.3	57	0.033	-291.99371
Centrifugation	312	24.1	55	0.103	115.20539
Centrifugation	625	23.8	57	0.169	499.13597
Centrifugation	1250	24.3	56	0.329	1429.87677
Centrifugation	2500	24.5	57	0.524	2564.21712
Centrifugation	5000	23.2	58	0.948	5030.68024
Centrifugation	10000	24.1	55	1.945	10830.35885
Centrifugation	20000	24.0	57	3.437	19509.51681
Centrifugation	316	23.0	57	0.107	138.47391
Centrifugation	562	24.2	55	0.147	371.15911
BPS	562	23.9	55	0.152	400.24476
BPS	0	24.5	57	0.047	-210.55389
BPS	316	23.4	57	0.112	167.55956
BPS	562	23.1	57	0.158	435.14754
BPS	0	23.0	57	0.037	-268.72519
Centrifugation	1000	24.1	55	0.296	1237.91148
BPS	1000	24.2	56	0.320	1377.5226
BPS	562	23.5	57	0.143	347.89059
BPS	1000	22.9	57	0.269	1080.84897
Centrifugation	562	24.2	56	0.155	417.69615
BPS	1000	23.7	57	0.303	1278.63139
BPS	316	22.7	57	0.129	266.45077
Centrifugation	562	23.2	57	0.149	382.79337
Centrifugation	1000	23.2	55	0.288	1191.37444
BPS	316	22.7	57	0.099	91.93687
BPS	0	24.0	56	0.032	-297.81084
Centrifugation	316	22.8	57	0.105	126.83965
Centrifugation	1000	23.7	58	0.279	1139.02027
Centrifugation	316	22.8	56	0.118	202.46234

Figure S3. Analysis of the structure of the residuals for the standard S100B detection model using ELISA.

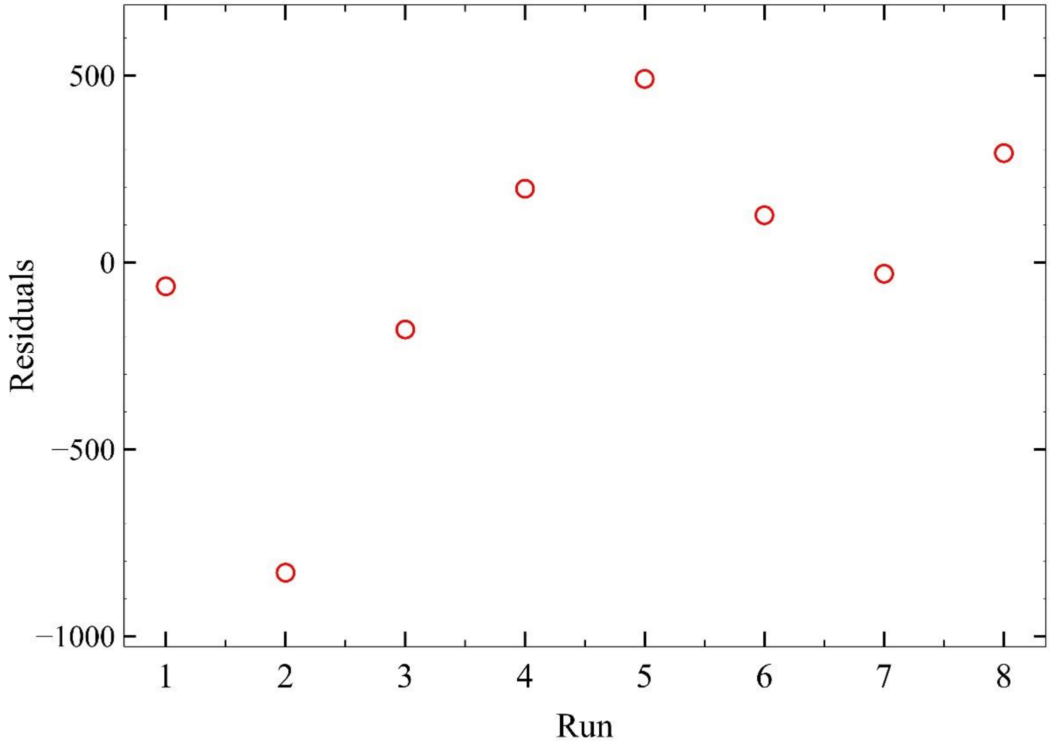

Table S4. Suitability analysis of the ELISA S100B detection standard model.

Coefficients

	Least Squares	Standard	T	
Parameter	Estimate	Error	Statistic	P-Value
Intercept	-483.959	198.46	-2.43857	0.0406
Slope	5817.13	136.473	42.6248	0.0000

Analysis of Variance

Source	Sum of Squares	Df	Mean Square	F-Ratio	P-Value
Model	3.35311E8	1	3.35311E8	1816.88	0.0000
Residual	1.10732E6	6	184553.		
Total (Corr.)	3.36418E8	7			

```
Correlation Coefficient = 0.998353
R-squared = 99.6709 percent
R-squared (adjusted for d.f.) = 99.616 percent
Standard Error of Est. = 429.597
Mean absolute error = 276.284
Durbin-Watson statistic = 1.35445 (P=0.2184)
Lag 1 residual autocorrelation = 0.282416
```

Table S5. Pearson correlation between OD and temperature and humidity.

```
Pearson's product-moment correlation
cor.test(DOPP,temp)
data:  DOPP and temp
t = 0.15285, df = 10, p-value = 0.8816
alternative hypothesis: true correlation is not equal to 0
95 percent confidence interval:
 -0.5406022  0.6054055
sample estimates:
       cor
0.04827767

> cor.test(DOPP,humidity)

      Pearson's product-moment correlation

data:  DOPP and humidity
t = -0.24829, df = 10, p-value = 0.8089
alternative hypothesis: true correlation is not equal to 0
```

```
95 percent confidence interval:

 -0.6241394  0.5189379

sample estimates:

        cor

-0.07827579
```

Figure S4. Analysis of the structure of the residuals for the t-test of conventional and paper-based BPS runs for measuring S100B using ELISA.

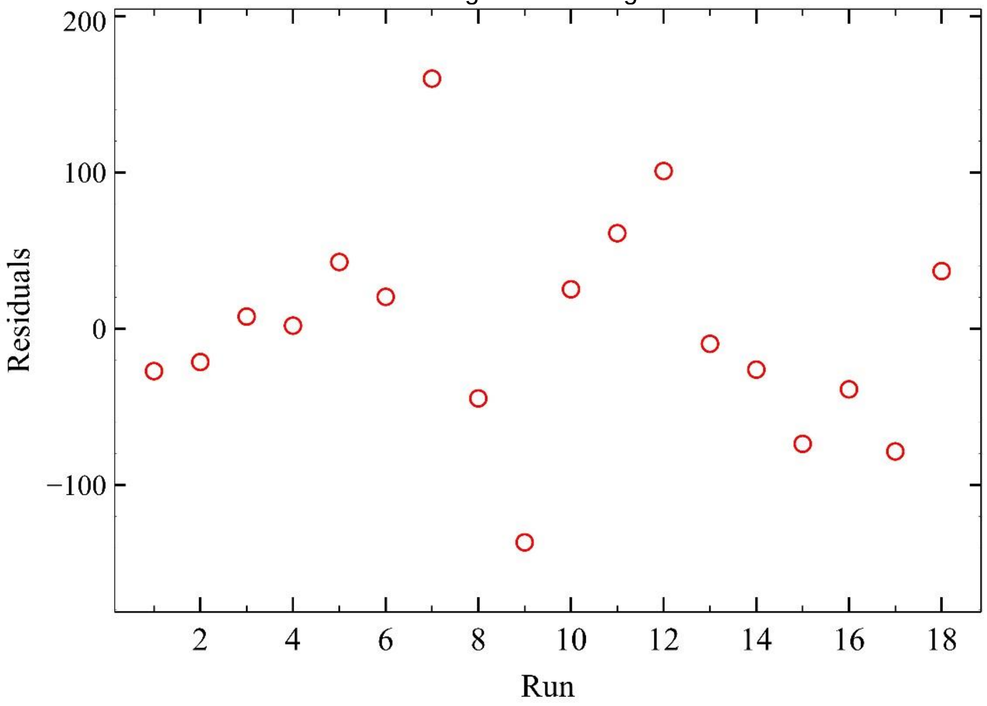

Supplement: Supplementary file 1 [file micromachines-13-00706-s001.zip › micromachines-1687643-SI.pdf]
